# Acid Sphingomyelinase Inhibition Prevents Development of Sepsis Sequelae in the Murine Liver

**DOI:** 10.1038/s41598-017-11837-2

**Published:** 2017-09-27

**Authors:** Ha-Yeun Chung, C. Julius Witt, Nayla Jbeily, Jorge Hurtado-Oliveros, Benjamin Giszas, Amelie Lupp, Markus H. Gräler, Tony Bruns, Andreas Stallmach, Falk A. Gonnert, Ralf A. Claus

**Affiliations:** 1 Center for Sepsis Control and Care, Jena University Hospital, Jena, 07747 Germany; 2 Department of Anesthesiology and Intensive Care, Jena University Hospital, Am Klinikum 1, Jena, 07747 Germany; 3 Institute of Pharmacology and Toxicology, Jena University Hospital, Jena, 07747 Germany; 4 Department of Internal Medicine IV (Gastroenterology, Hepatology and Infectious Diseases), Jena University Hospital, Jena, 07747 Germany; 5 Present Address: Hans-Berger Department of Neurology, Jena University Hospital, Jena, 07747 Germany

## Abstract

The molecular mechanisms of maladaptive response in liver tissue with respect to the acute and post-acute phase of sepsis are not yet fully understood. Long-term sepsis survivors might develop hepatocellular/hepatobiliary injury and fibrosis. Here, we demonstrate that acid sphingomyelinase, an important regulator of hepatocyte apoptosis and hepatic stellate cell (HSC) activation, is linked to the promotion of liver dysfunction in the acute phase of sepsis as well as to fibrogenesis in the long-term. In both phases, we observed a beneficial effect of partial genetic sphingomyelinase deficiency in heterozygous animals (smpd1^+/−^) on oxidative stress levels, hepatobiliary function, macrophage infiltration and on HSC activation. Strikingly, similar to heterozygote expression of SMPD1, either preventative (p-smpd1^+/+^) or therapeutic (t-smpd1^+/+^) pharmacological treatment strategies with desipramine – a functional inhibitor of acid sphingomyelinase (FIASMA) – significantly improved liver function and survival. The inhibition of sphingomyelinase exhibited a protective effect on liver function in the acute-phase, and the reduction of HSC activation diminished development of sepsis-associated liver fibrosis in the post-acute phase of sepsis. In summary, targeting sphingomyelinase with FDA-approved drugs is a novel promising strategy to overcome sepsis-induced liver dysfunction.

## Introduction

Sepsis continues to be a serious problem worldwide with mortality rates reaching up to 18% due to consecutive organ failure and maladaptive host response^1,2^. The development of organ dysfunction resulting in multiple organ failure is a hallmark of the disease continuum which remains one major cause for poor outcome of these patients^3^. In particular, hepatic excretory dysfunction is an early and common consequence of sepsis^4^. In addition to challenges in managing the acute phase of the overwhelming host response to infection, long-term consequences of sepsis in survivors are still an underestimated issue compromising quality of life and resulting in an increased mortality risk following hospital discharge^5^. Previous studies have demonstrated that sepsis leads to increased liver-stiffness^6^ and prolonged sepsis-associated cholestasis with associated mortality^7,8^. The latter even with the risk to result in progressive sclerosing cholangitis^9^. These phenomena are also mimicked in a long-term murine model of sepsis where fibrosis of the liver subsequent to hepatic stellate cell (HSC) activation was shown in survivors^10^.

Among the plethora of pathophysiological changes in sepsis, the enhanced activity of the stress responsive enzyme sphingomyelin-phosphodiesterase 1 (SMPD1) might function as a promising target for therapeutic interventions^11–13^. Previous data suggest a positive association between an increased concentration as well as activity in plasma and disease severity allowing discrimination of outcome of these patients^14^. Furthermore, the regulation of this enzyme by a functional inhibitor improved either the survival of mice in an endotoxemia model^11^ or overcoming from cardiomyopathy due to polymicrobial sepsis^15^. SMPD1 is responsible for the fast and transient breakdown of membrane-embedded sphingomyelin to ceramide which in turn accumulates in lipid rafts of the outer layer of the cell membrane thus reorganizing protein complexes and supporting downstream signal transduction of inflammatory pathways^16,17^. SMPD1 is necessary for numerous signaling pathways controlling proliferation, autophagy, differentiation and apoptosis^18–20^. On a molecular level, there is also evidence of a direct regulation of the activation process of HSC into activated liver myofibroblasts (aLMF) by SMPD1 controlling extracellular matrix (ECM) production and deposition. The inhibition of SMPD1 abrogates the transdifferentiation of primary mouse hepatic stellate cells *in vitro* and reduces fibrogenesis in an *in vivo* model of bile-duct ligation^21^. Moreover, numerous studies have also provided a direct impact of SMPD1 activity on hepatocyte damage *in vitro* as well as *in vivo* ^22,23^. In hepatocytes of SMPD1 deficient animals, the total loss of enzyme function protects these cells from Fas- and TNF-α-mediated apoptosis^24,25^.

In order to characterize the effects of SMPD1 in the setting of acute- and post-acute liver dysfunction following the host response during sepsis, we opted for different inhibition strata: As a model of pharmacological inhibition, we preventively treated smpd1^+/+^ animals (p-smpd1^+/+^) for seven days prior to the induction of sepsis with desipramine, a well-known functional inhibitor of SMPD1 (FIASMA)^26^. Furthermore, we established a therapeutic strategy treating smpd1^+/+^ mice (t-smpd1^+/+^) with desipramine 6 hours following induction of polymicrobial sepsis, in order to evaluate a potential role of its therapeutic use. Additionally, we compared these animals with septic smpd1^+/+^ littermates as well as with mice partially deficient for the enzyme (smpd1^+/−^). At three time points representing different phases of sepsis-associated liver damage and fibrogenesis (24 hours, 3 and 28 days), we monitored the role of SMPD1 in the maintenance of hepatic (dys)function during the acute-phase and in the prevention of hepatic fibrosis during the post-acute phase of sepsis.

## Results

### Detrimental role of SMPD1 in liver function following polymicrobial sepsis

**Figure 1:**
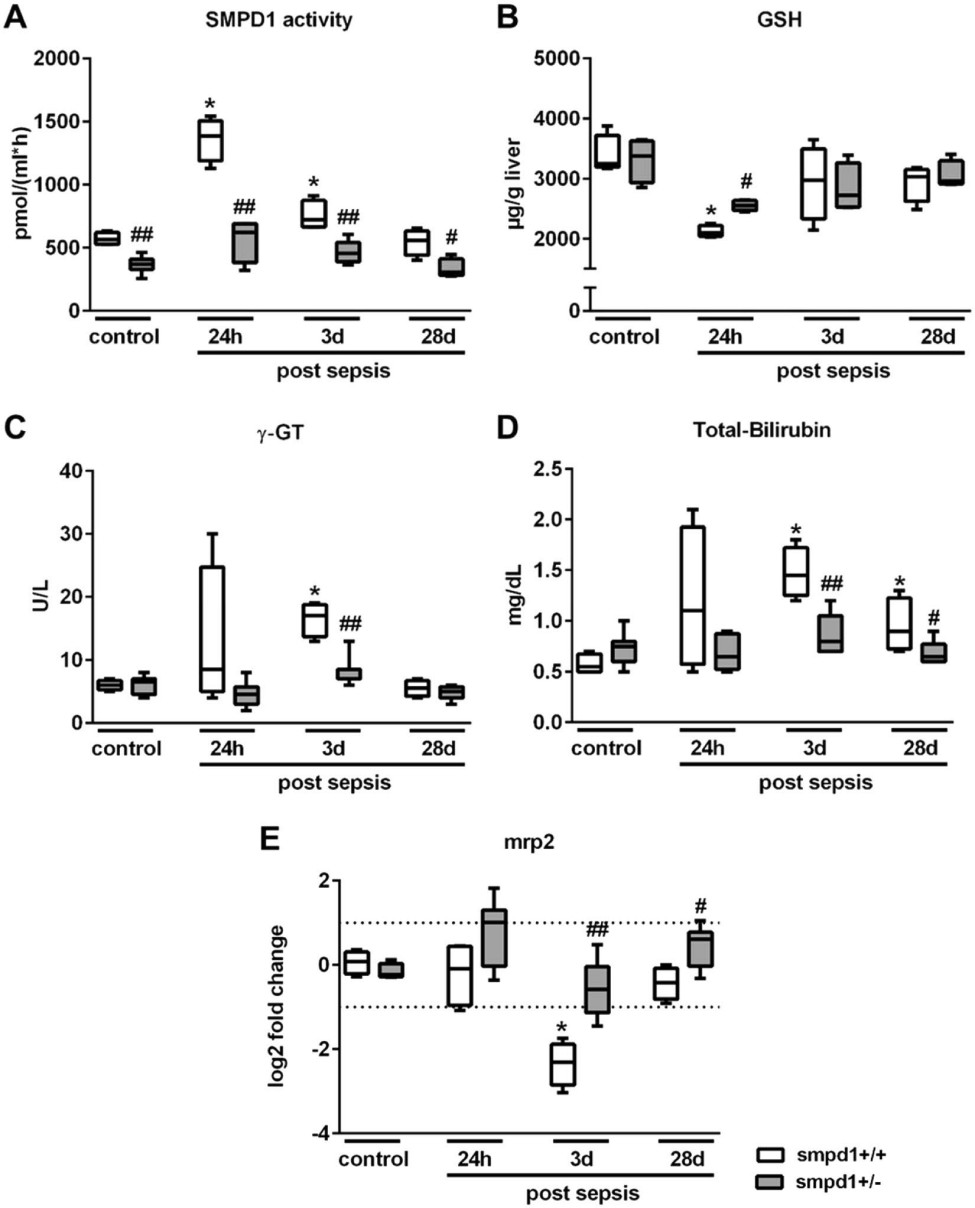
Increased SMPD1 activity is highly associated with liver dysfunction in polymicrobial sepsis. Polymicrobial sepsis was induced in smpd1^+/+^ and smpd1^+/−^ mice by peritoneal contamination and infection to determine (**A**) serum SMPD1 activity (Fig. 1A; n = 4 smpd1^+/+^, n = 8 smpd1^+/−^), (**B**) liver glutathion (GSH) levels (Fig. 1B; n = 4 smpd1^+/+^, n = 4 smpd1^+/−^), (**C**) γ-GT, (**D**) serum total bilirubin (Fig. 1C,D; n = 4 smpd1^+/+^, n = 8 smpd1^+/−^) as well as (**E**) hepatic transcriptional expression of Mrp2 (Fig. 1E; n = 4 smpd1^+/+^, n = 8 smpd1^+/−^) at three different time points (24 hours, 3 and 28 days). ^#^p < 0.05; ^##^p < 0.01 vs. corresponding smpd1^+/+^ control; *p < 0.05; **p < 0.01 vs. baseline (MWU-test). Transcriptional expression is normalized to reference gene (Actb) and shown as log2 fold changes. Cut off values were set at ± 1, representing a variation of biological significance (dotted lines).

Previous studies have demonstrated that the increased activity of the conserved stress responsive enzyme SMPD1 is associated with impaired outcome in septic patients and animal models^11,27^. Measurement of the SMPD1 activity in serum samples of smpd1^+/+^ littermates revealed an increased activity at 24 hours and 3 days following sepsis induction, whereas at day 28 the SMPD1 activity did not differ from that in sham-treated animals. In contrast, smpd1^+/−^ animals demonstrated significant lower activity levels at all time points as compared to smpd1^+/+^ mice (Fig. 1A). Furthermore, smpd1^+/+^ animals had depleted glutathion (GSH) levels in liver homogenates at 24 hours following sepsis induction indicating noticeable oxidative stress. The partial genetic inhibition of the enzyme significantly improved oxidative stress levels in the acute phase of sepsis (Fig. 1B). In addition, smpd1^+/−^ animals had lower surrogates of liver dysfunction as represented by γ-GT, total-bilirubin and the transcriptional expression of Mrp2 levels at day 3 following sepsis as compared to wild-type smpd1^+/+^ animals (Fig. 1C–E). These results demonstrate that the stress responsive enzyme SMPD1 is an essential key player in the development of hepatic dysfunction during polymicrobial sepsis.

### Inhibition of SMPD1 reveals its anti-inflammatory capacity in sepsis

**Figure 2:**
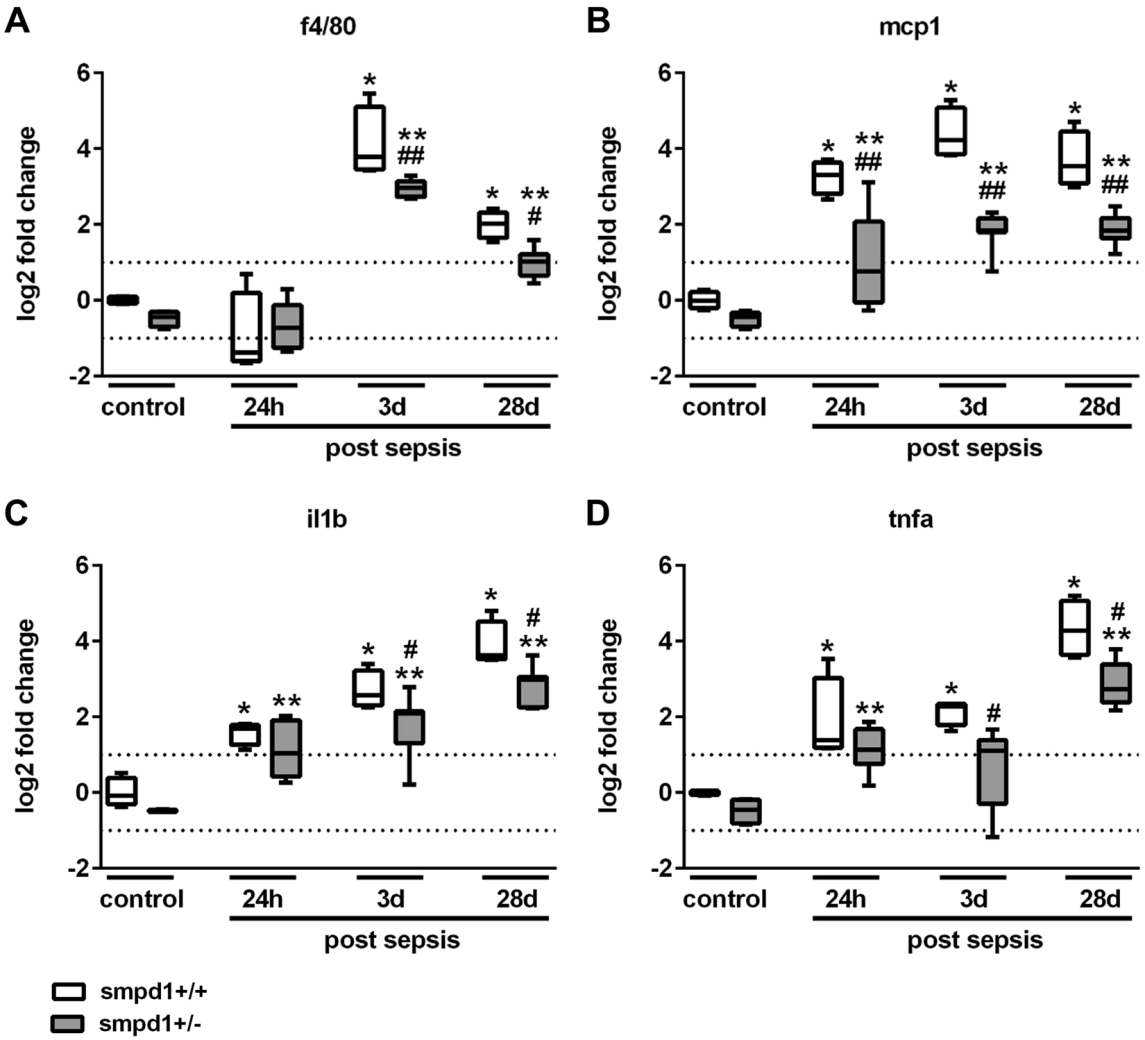
SMPD1 drives hepatic inflammation in polymicrobial sepsis. Hepatic gene expression of (**A**) f4/80, (**B**) Ccl2/mcp1, (**C**) Il1b and (**D**) Tnfa was determined at three different time points following polymicrobial sepsis (24 hours, 3 and 28 days; n = 4 smpd1^+/+^, n = 8 smpd1^+/−^) to evaluate the impact of SMPD1 on hepatic inflammation. Transcriptional expression is normalized to reference gene (Actb) and shown as log2 fold changes. Cut off values were set at ± 1, representing a variation of biological significance (dotted lines). ^#^p < 0.05; ^##^p < 0.01 vs. corresponding smpd1^+/+^ control; *p < 0.05; * *p < 0.01 vs. baseline (MWU-test).

To further evaluate the hepatic inflammatory response, we assessed the expression of f4/80, a marker of activated macrophages, and of cytokines characteristically increased during the course of sepsis, such as Ccl2/mcp1, Tnfa as well as Il1b in liver homogenates of smpd1^+/+^ mice. Consistent with a detrimental role for SMPD1 in the hepatic inflammatory response during sepsis, these markers of hepatic inflammation were significantly lower in smpd1^+/−^ animals as compared to smpd1^+/+^ mice (Fig. 2A–D).

### Acid sphingomyelinase controls HSC transdifferentiation and hepatic fibrosis in long-term survivors of sepsis

**Figure 3:**
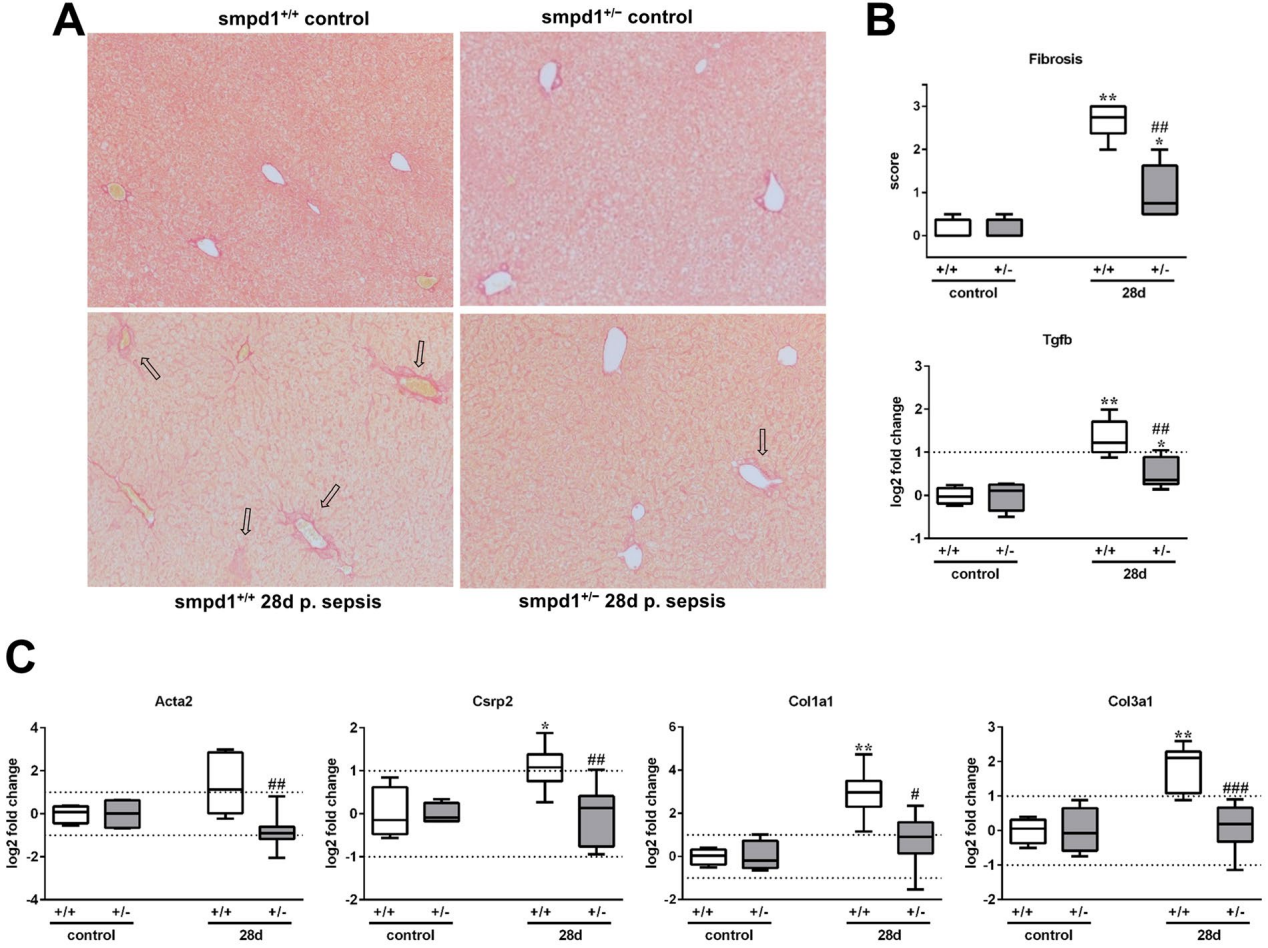
Reduced HSC activation and hepatic fibrosis in smpd1^+/−^ mice after polymicrobial sepsis. In long-term survivors (28 days following sepsis induction) (**A**) representative Sirius Red stainings, (**B**) hepatic fibrosis scores and Tgfb expression (Fig. 2A/B; n = 4 smpd1^+/+^, n = 7 smpd1^+/−^) and (**C**) hepatic gene expression of fibrosis-associated genes (Fig. 2C; n = 4 smpd1^+/+^, n = 8 smpd1^+/−^) were determined. Transcriptional expression is normalized to reference gene (Actb) and shown as log2 fold changes. Cut off values were set at ± 1, representing a variation of biological significance (dotted lines). ^#^p < 0.05; ^##^p < 0.01 vs. corresponding smpd1^+/+^ control; *p < 0.05; * *p < 0.01 vs. baseline (MWU-test).

HSC activation, which is known to be controlled by SMPD1^21^, ultimately resulted in ECM deposition in a long term murine sepsis model^10^. At day 28 following sepsis Sirius Red staining revealed fibrotic changes in the parenchyma, around the portal tract and central veins in smpd1^+/+^ mice, whereas smpd1^+/−^ animals displayed less fibrotic areas in the parenchyma and around the portal tracts (Fig. 3A). Semi-quantitative scoring of ECM deposition attested a strong increase in smpd1^+/+^ animals, with a less pronounced collagen deposition in smpd1^+/−^ littermates (Fig. 3B). Hepatic gene expression of *α-smooth muscle actin 2* (Acta2) and *cysteine and glycine rich protein 2* (Csrp2), both of which are well established measures of activated liver myofibroblasts, confirmed a significant increase in smpd1^+/+^ animals, which was not evident in smpd1^+/−^ animals (Fig. 3C). The expression of Tgfb was significantly higher in smpd1^+/+^ as compared in smpd1^+/−^ littermates (Fig. 3B). Consistently, hepatic expression of different collagens (Col1a1, Col3a1) was found to be increased 28 days following septic insult in smpd1^+/+^ but ameliorated in smpd1^+/−^ animals (Fig. 3C). These results suggest SMPD1 as a key player in the control of post-inflammatory HSC activation and hepatic fibrogenesis as a long-term sequelae of sepsis.

### Pharmacological inhibition of SMPD1 by desipramine results in improved liver function and decreased hepatic inflammatory response during polymicrobial sepsis

**Figure 4:**
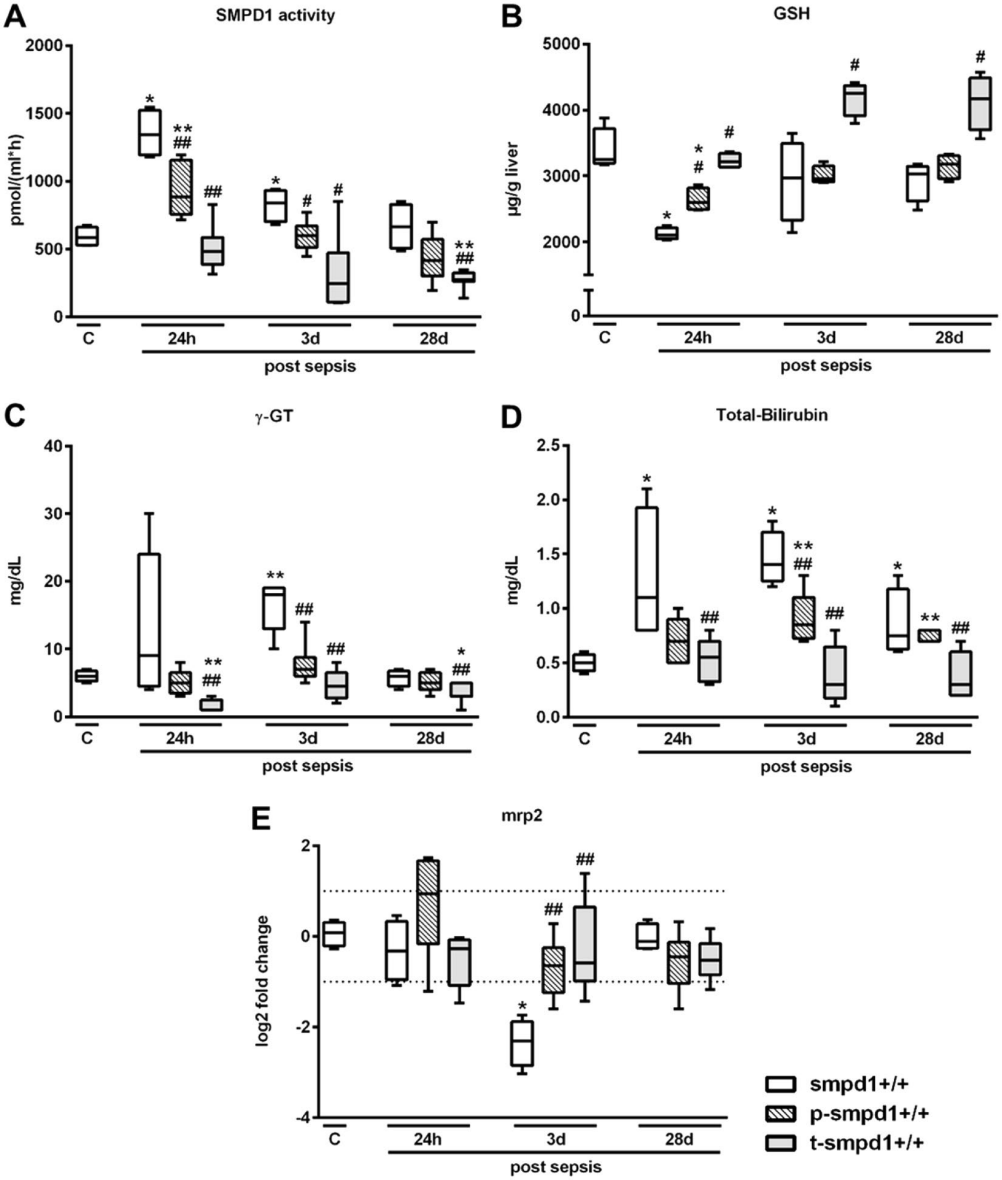
Preventative and therapeutic desipramine treatment abrogates sepsis-induced liver dysfunction in smpd1^+/+^ mice. Smpd1^+/+^ mice were treated either 7 days prior (preventative, p-smpd1^+/+^) or 6 hours (therapeutic, t-smpd1^+/+^) following sepsis induction with desipramine. (**A**) Serum SMPD1 activity (Fig. 4A; n = 4 smpd1^+/+^, n ≥ 6 p-smpd1^+/+^, t-smpd1^+/+^), (**B**) liver glutathion (GSH) levels (n = 4 smpd1^+/+^, n = 4 p-smpd1^+/+^, t-smpd1^+/+^), (**C**) γ-GT, (**D**) total serum bilirubin (n = 4 smpd1^+/+^, n ≥ 6 p-smpd1^+/+^, t-smpd1^+/+^) and (**E**) hepatic gene expression of Mrp2 (Fig. 4E; n = 4 smpd1^+/+^, n ≥ 5 p-smpd1^+/+^, t-smpd1^+/+^) are shown. Transcriptional expression is normalized to reference gene (Actb) and shown as log2 fold changes. Cut off values were set at ± 1, representing a variation of biological significance (dotted lines). ^#^p < 0.05; ^##^p < 0.01 vs. corresponding smpd1^+/+^ control; *p < 0.05; * *p < 0.01 vs. baseline (MWU-test).

**Figure 5:**
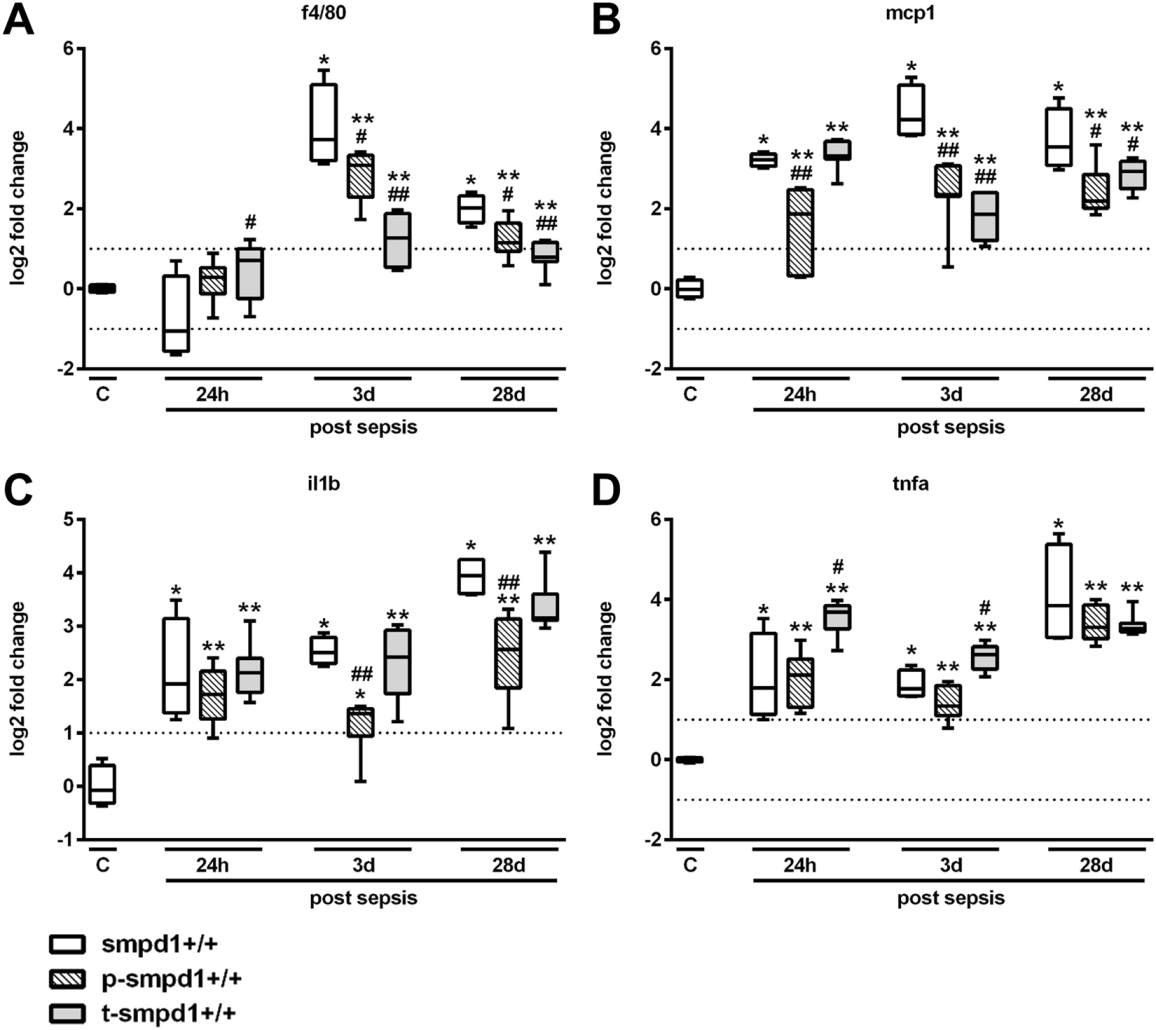
Preventative treatment strategy as well as therapeutic treatment diminishes hepatic inflammatory response during polymicrobial sepsis. Hepatic gene expression of (**A**) f4/80, (**B**) Ccl2/mcp1, (**C**) Il1b and (**D**) Tnfa was determined at three different time points following polymicrobial sepsis (24 hours, 3 and 28 days; n = 4 smpd1^+/+^, n = 8 p-smpd1^+/+^, t-smpd1^+/+^) to evaluate the impact of SMPD1 on hepatic inflammation following either preventative (p-smpd1^+/+^) or therapeutic (t-smpd1^+/+^) treatment with desipramine during sepsis. Transcriptional expression is normalized to reference gene (Actb) and shown as log2 fold changes. Cut off values were set at ± 1, representing a variation of biological significance (dotted lines). ^#^p < 0.05; ^##^p < 0.01 vs. corresponding smpd1^+/+^ control; *p < 0.05; **p < 0.01 vs. baseline (MWU-test).

FIASMAs are known to have beneficial effects on numerous hepatic pathologies, such as Wilson’s disease or alcohol-induced liver cirrhosis^23,28^. Therefore, we tested both, a preventative and a therapeutic treatment strategy with the FIASMA desipramine, to alleviate hepatic dysfunction and liver injury after sepsis. SMPD1 was significantly diminished in smpd1^+/+^ animals preventatively or therapeutically treated with desipramine (Fig. 4A). Furthermore, GSH levels (Fig. 4B), and surrogates of liver dysfunction (Fig. 4C–E) were significantly improved 24 hours after sepsis induction as compared to controls. These results confirm observations from heterozygous animals where treatment with desipramine in a preventative as well as in a therapeutic fashion results in a suppression of SMPD1 activation and an improvement of liver dysfunction during systemic inflammation. Mice preventatively treated with the functional inhibitor desipramine demonstrated significantly lower expression levels of f4/80, Ccl2/mcp1, Tnfa and Il1b in liver homogenates as compared to controls (Fig. 5). Despite the normalization of functional parameters, markers of inflammation were found to be increased, but diminished compared to smpd1^+/+^ controls, indicating a lower extent of chronic exacerbation of inflammation. Comparable results were obtained with respect to therapeutic treatment with desipramine (Fig. 5).

### Pharmacological inhibition of SMPD1 reduced hepatic fibrosis after sepsis

**Figure 6:**
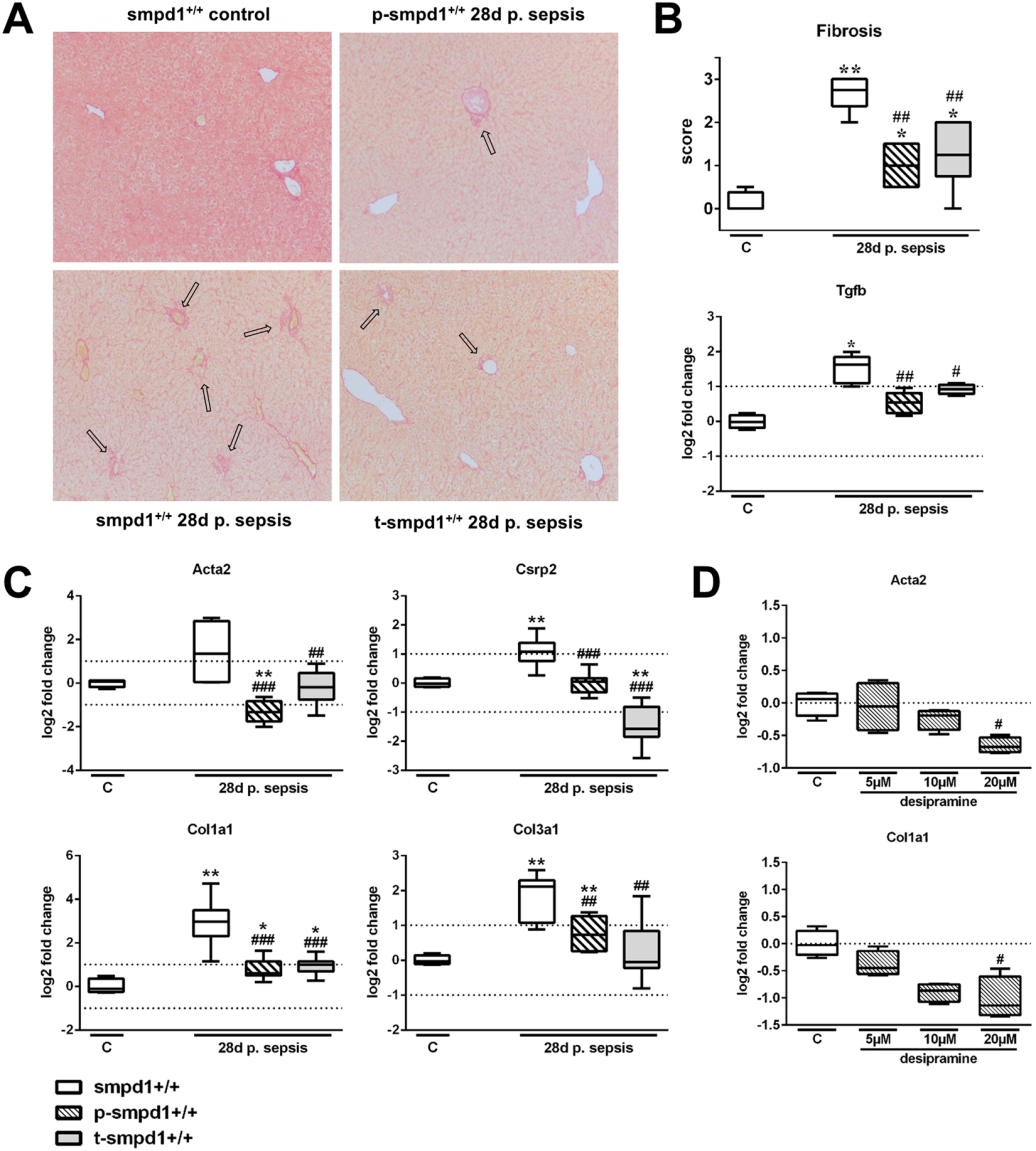
Pharmacological inhibition of SMPD1 results in reduced HSC activation and reduced hepatic fibrosis. In long-term survivors (28 days following sepsis induction) (**A**) representative Sirius Red stainings, (**B**) hepatic fibrosis scores and Tgfb expression (Fig. 6A/B; n = 4 smpd1^+/+^, n ≥ 5 p-smpd1^+/+^, t-smpd1^+/+^) and (**C**) hepatic gene expression of fibrosis-associated genes (Fig. 6C; n = 4 smpd1^+/+^, n = 8 p-smpd1^+/+^, t-smpd1^+/+^) were determined. Transcriptional expression is normalized to reference gene (Actb) and shown as log2 fold changes. (**D**) Furthermore, stimulation for 24 hours with different desipramine concentrations (5 µM, 10 µM, 20 µM) of LX-2 cells were performed presenting dose dependent inhibition of ACTA2 as well as COL1A1 expression rate (Fig. 6D; n = 4). Transcriptional expression is normalized to reference genes (GAPDH) and presented in log2 fold changes. Cut off values were set at ± 1, representing a variation of biological significance (dotted lines). ^#^p < 0.05; ^##^p < 0.01 vs. corresponding smpd1^+/+^ control; *p < 0.05; * *p < 0.01 vs. baseline (MWU-test).

Since it is known that functional inhibitors of SMPD1, such as imipramine, are capable of reducing hepatic fibrosis, we were interested whether desipramine, also a functional inhibitor, is capable to reduce hepatic fibrosis occurring in sepsis survivors as a long-term sequela. Collagen deposition was reduced in desipramine-treated animals 28 days after the onset of sepsis according to Sirius Red staining (Fig. 6A,B). Consistently, the hepatic expression of Acta2, Csrp2, Col1a1 and Col3a1 was improved by both treatment strategies indicating reduced activation of liver myofibroblasts and reduced expression of extracellular matrix (Fig. 6C). To confirm a protective effect of desipramine on HSC activation *in vitro*, we treated LX-2 cells with desipramine in different concentrations for 24 hours. The stimulation with desipramine resulted in a downregulation of ACTA2 as well as COL1A1 expression in a dose-dependent manner (Fig. 6D).

### SMPD1 inhibition improves survival following polymicrobial sepsis

**Figure 7:**
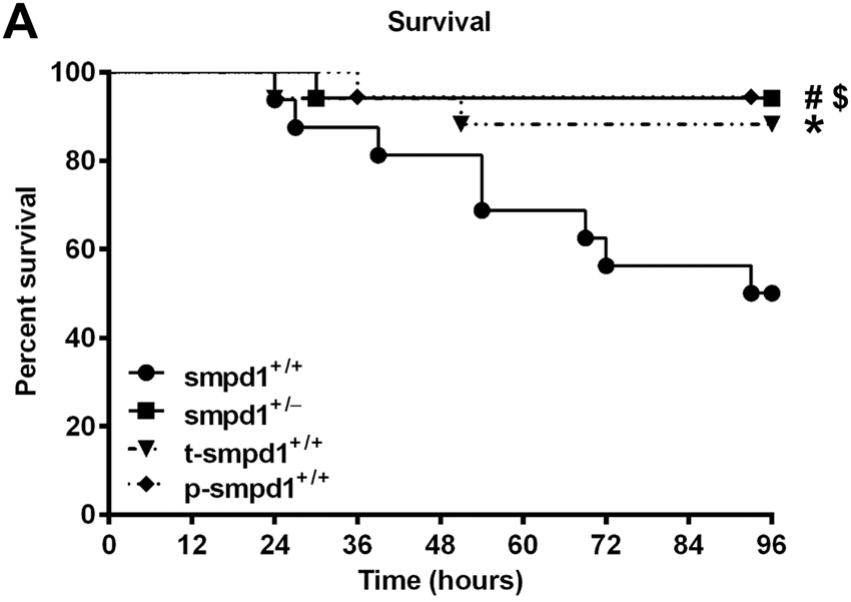
Partial genetic and pharmacological inhibition of SMPD1 improves survival in polymicrobial sepsis. Kaplan-Meier survival analysis [96 hours] with respect to SMPD1 controlled either by genotype (smpd1^+/−^) or by treatment (p-smpd1^+/+^, t-smpd1^+/+^) following polymicrobial sepsis. Randomly selected smpd1^+/+^ mice were pretreated 7 days prior or 6 hours following sepsis induction with desipramine and compared to smpd1^+/+^, smpd1^+/−^ littermates. Data were obtained from at least n ≥ 15 of each stratum. Statistical analyses were performed using log-rank test for trend (p = 0.0152); subsequently performed log-rank test attested significant differences between smpd1^+/+^ and smpd1^+/−^ (^**#**^p = 0.0057); smpd1^+/+^ and p-smpd1^+/+^ (^**$**^p = 0.0042); smpd1^+/+^ and t-smpd1^+/+^ (*p = 0.0245).

As a previous study has already demonstrated a beneficial role of SMPD1 inhibition for survival in a murine model of sterile endotoxemia^11^, we tested the hypothesis if this holds true in a polymicrobial sepsis model of peritoneal contamination and infection (PCI) with subsequent antibiotic rescue. As shown in Fig. 7, the overall survival revealed a rate of 50% in smpd1^+/+^ animals in an observation period of 96 hours. In contrast, smpd1^+/−^ animals displayed a significant reduction of mortality following polymicrobial sepsis with similar findings for preventative treatment with desipramine 7 days prior to sepsis induction as well as therapeutic treatment with desipramine 6 hours following sepsis induction.

## Discussion

This study provides first evidence of a detrimental role of the conserved stress responsive enzyme SMPD1 in the development of sepsis-induced liver dysfunction and fibrosis, which both affect the outcome from sepsis and the quality of life in sepsis survivors. We show that reducing SMPD1 activity, either by partial genetic deficiency or mostly important by preventative or therapeutic pharmacological inhibition, is beneficial for liver function in the acute phase of sepsis and for preventing inflammation, HSC activation and hepatic fibrogenesis as a prolonged consequence of sepsis.

Altered SMPD1 activity has been linked to the pathogenesis of numerous disease entities such as alcoholic cirrhosis, non-alcoholic steatohepatitis, as well as cachexia and mortality in chronic heart failure^20,23,29^. Protective effects due to deficiency could be reversed by treatment with exogenously added ceramide^30,31^. In addition, *in vitro* treatment of rat hepatocytes with human placenta SMPD1 induced oxidative stress and initiated apoptosis^32^.

Moreover, an increased activity of secreted SMPD1 in plasma samples of septic patients correlates with disease severity and poor outcome, and its inhibition improved survival rate in a murine model of severe endotoxemia^11^, in a *Staphylococcus aureus* or polymicrobial sepsis model^15,27^. In order to clarify the role of SMPD1 as a stress enzyme in sepsis-induced liver dysfunction, we used mice, which are partially deficient in the enzyme. It is controversially discussed whether it is appropriate to use this strain of genetically deficient mice in order to prove pathophysiological effects and cellular mechanisms based on the fact that mice compensate their total loss of enzyme function^33^. The complete knock-out of SMPD1 gene results in Niemann-Pick disease which is characterized by rapid central nervous degeneration alongside an impairment of liver function^34^. However, minimal residual enzymatic activity is sufficient to prevent Niemann-Pick disease and its neurological symptoms^35^. In line with these studies, we observed increased oxidative stress as well as elevated cytokine responses in knock-out animals^36^, which encouraged us to investigate mice heterozygous for SMPD1 (smpd1^+/−^) presenting with decreased but still residual SMPD1 activity levels. In addition to the beneficial effect of partial deficiency of SMPD1 in a bile duct ligation^21^ as well as in a Fas-mediated apoptosis model^25^, we infer from our sepsis model that residual activity of SMPD1 in smpd1^+/−^ mice is sufficient to prevent maladaptive effects caused by the overwhelming activity in smpd1^+/+^ animals.

In this study smpd1^+/−^ mice had a favorable phenotype resulting in improved liver function and reduced hepatic oxidative stress in the course of sepsis. In the case of sepsis-induced liver dysfunction there is still a broad discussion regarding its etiology and underlying molecular mechanism. One of these concepts denotes that immune cells, such as liver invading monocytes, contribute to the pathogenesis of sepsis-induced liver failure due to the overwhelming production of cytokines^37^. Consistent with the fact that SMPD1 is a key player in the regulation of macrophage differentiation and cytokine secretion, we demonstrate that activation of these cells and further cytokine expression is a function of SMPD1 activity. Furthermore, reduced hepatic fibrogenesis as a long-term sequela of sepsis, was proven by the expression profile of Acta2^38^ and Csrp2^39^, which are well established markers for activated liver myofibroblasts. Our data revealed that in smpd1^+/−^ mice the number of aLMF was reduced which might be mirrored in a diminished deposition of ECM in the liver. In fact, the expression profile of two components of ECM (Col1a1, Col3a1) was found reduced on both, the molecular and the histological level. Our data are in line with a previous study from Moles *et al*. identifying SMPD1 as a key player HSC activation and hepatic fibrogenesis^21^.

In our study we elaborated causes and consequences of sepsis-induced fibrogenesis and its modulation by approved drugs. For that issue we analyzed samples from tissue homogenates and sections from representative locations. To solve the more specific question, whether there is not only a change in (trans-)activation state of hepatic stellate cells but also a variation in number (*i.e*. proliferation) or even a phenotypic dissociation of stellate cells affected, one might consider the temporal-spatial conditions of liver tissue undergoing adaptive response, but also the presence of granulomatous inflammation as a response to persistent inflammation/infection^10^ due to inflammasome activation in stellate cells^40^. Then, the generation and distribution of tissue abundant abscesses and the status of inflammasome activation might be correlated with a putative phenotypic dissociation of stellate cells.

Our data demonstrate that desipramine, a functional inhibitor of this stress enzyme, is capable of alleviating elevated enzyme activity during the host response towards in order to reduce sepsis-induced hepatic injury and fibrogenesis. This observation is of major interest since the used inhibitor is a member of a class of drugs (FIASMA) all of which regulate the activity of SMPD1 and have been used in daily clinical care for decades in treatment of neurological disorders^41,42^. A series of studies have provided evidence that the functional inhibition of SMPD1 improves cellular stress responses, reduces oxidative stress and improves viability *in vivo* ^15^ or *in vitro* ^30,32,43,44^. In our study, we expand the knowledge of a beneficial effect of SMPD1 inhibition by treating animals with desipramine 7 days prior and 6 hours following to severe infection, resulting in decreased SMPD1 activity thus followed by improved sepsis-induced oxidative stress. It is known that GSH belongs to intracellular antioxidant defense strategies and plays an important role in the balance of oxidants and antioxidants^45^. Interestingly, desipramine was demonstrated to restore glutathione levels^46^ and to further possess anti-inflammatory capacity^47^. These data are supported by the fact that p-smpd1^+/+^ as well as t-smpd1^+/+^ animals revealed less pronounced markers of inflammation as well as liver dysfunction such as total-Bilirubin and γ-GT as well as oxidative stress compared to smpd1^+/+^ animals. Comparison of smpd1^+/−^ mice to p-smpd1^+/+^ and t-smpd1^+/+^ animals indicates similarities or minimal differences in all parameters, especially with respect to chronic exacerbation of inflammation. Of note, in our experimental setting, no immune-suppressive effect of desipramine treatment was observed^48^.

In addition, we show an improved survival rate after polymicrobial sepsis in desipramine-treated smpd1^+/+^ mice as well as in smpd1^+/−^ mice.

With the aim of fostering the excretory capacity also the functionality of hepatocytes and cholangiocytes as well as the restitution of excretory function in presence of inhibitors of sphingomyelinase needs enhancing perception for upcoming experimental work. Primary hepatocytes for *ex-vivo* experimentation or animal experiments with distinct (mixed) genotypes might function as a reliable tool to get further insights into the complex regulatory mechanisms of membrane integration of MDR2, its cytoskeletal anchoring (phosphorylation/ dephosphorylation), or ultimately reorganization upon inflammation in membranous subdomains characterized by a specific lipid composition^4^. Since the lipid mediator ceramide is known to affect and also to control mostly all of these key events, the exploration of the (local) kinetic) and dynamic of ceramide generation and its accumulation is of great interest, especially with respect to MDR2-function.

Overall, results from our study support the notion that an increased activity level of SMPD1 displays a detrimental role with respect to regulation of liver function in both the acute and post-acute phase of sepsis. The decreased, but residual SMPD1 activity in smpd1^+/−^, p-smpd1^+/+^ and t-smpd1^+/+^ mice are even sufficient for regulation of adaptive mechanisms in the liver during host response. Finally, our data highlights the favorable effect of functional SMPD1 inhibition, i.e. by desipramine, in order to protect the liver from hepatocyte damage *in vivo* which is underlined by obtained data from smpd1^+/−^ animals. Furthermore, the pharmacological inhibitor diminished the activation of HSC and prevented fibrogenesis in survivors as a prolonged consequence of severe infection. We are proposing a concept of a causative therapeutic strategy by evaluation of a novel target. As these SMPD1 inhibiting drugs are FDA-approved drug, which are frequently prescribed for neurological disorders, observational studies in well-defined patients could unravel, whether FIASMA use is associated with reduced liver injury in patients with sepsis.

## Material and Methods

### Cell culture experiments

An immortalized hepatic stellate cell line (LX-2)^49^ of human origin, was cultured under standard conditions (1% heat inactivated fetal calf serum [FCS] in Dulbecco’s Modified Eagle Medium (DMEM) media, 5% CO_2_). Cells were treated 24 hours with different concentrations of desipramine (5 µM, 10 µM, 20 µM) prior to stimulation with cytokine mix (TNF-α, IFN-γ, IL-1β [each: 10 ng/mL] and 100 ng/mL of endotoxin) for 24 hours as described previously^50,51^.

### Animals

All experiments were performed in accordance with the German legislation on protection of animals and with approval of the local animal welfare committee (Thueringer Landesamt fuer Lebensmittelsicherheit und Verbraucherschutz, 02-009/12). We compared smpd1^+/−^ animals with a partial deficiency in SMPD1 function to smpd1^+/+^ littermates^52^. For each experiment, similar proportions of male and female gender were randomly selected at the age of 8–12 weeks (mean B.W. 22.2 g). Animals were housed under controlled/standardized day-night conditions (12 h/12 h) at room temperature (23 ± 1 °C, 30%–60% environmental humidity), received a standard diet and water ad libitum and were allowed to adapt to laboratory conditions for at least 2 days prior to the start of the experiments.

### Desipramine treatment and sepsis mode

Smpd1^+/+^ animals were treated 7 days prior or 6 hours following sepsis induction with desipramine-hydrochloride (isotonic saline solution, 20 mg/kg B.W.) every 24 hours subcutaneously which was continued daily up to euthanasia. Sepsis was induced by the standardized and established peritoneal contamination and infection approach^53^. Briefly, intraperitoneal injection of fecal slurry (diluted 1:4 in saline solution) was performed (3.0 µl/g B.W.) into the right lower quadrant of the abdomen with a 21-gauge cannula. Without supportive treatment, severity of the insult results in an approximately 100% mortality rate within 48 hours (data not shown). However, administration of antibiotics and volume resuscitation rescued animals and adjusted survival rate to 50% (20 mg/kg B.W. of meropenem every 24 hours s.c. and 25 µl/g B.W. of physiological saline solution s.c. twice daily over 4 days, starting 6 hours following sepsis induction). In the post-acute phase (day 3) treatment was restrained to desipramine-HCl/vehicle administration once daily (isotonic saline solution, 20 mg/kg B.W.). Disease progression and outcome of mice during host response was continuously assessed using the clinical severity scores (CSS) (data not shown) as previously described^53^. Animals were deeply anesthetized before any procedure by isoflurane (2%) which was followed by harvesting tissues and whole blood (right-ventricular heart puncture).

### SMPD1 activity

Serum was collected from each stratum (smpd1^+/+^, smpd1^+/−^, p-smpd1^+/+^, t-smpd1^+/+^) at baseline, 24 h, 3 and 28 days following the insult. SMPD1 activity was determined by the hydrolysis of fluorescently labeled sphingomyelin (NBD-SM; Molecular Probes, Eugene, OR) as a substrate, chromatographic product separation, and image analysis as described previously^54,55^. Serum samples were diluted 1:10 with the incubation buffer (sodium acetate, pH 5.0) before analysis. Finally, the reaction mixture was composed from 20 μl diluted serum and the extraction was carried out using a SpeedVac concentrator Plus (Eppendorf, Hamburg, Germany).

### GSH

Glutathione in its reduced form (GSH) was determined according to Ellman^56^. Briefly, fresh frozen liver samples were homogenized with 11 volumes of 0.2 M sodium phosphate buffer (5 mM EDTA; pH 8.0) and 4 volumes of 25% metaphosphoric acid. Following centrifugation (12000 x g; 4 °C; 30 min), GSH was measured in the supernatants photometrically. For analysis, data were obtained from n = 4 for baseline and n = 4 animals following polymicrobial sepsis from each stratum (smpd1^+/+^, smpd1^+/−^, p-smpd1^+/+^, t-smpd1^+/+^).

### Laboratory markers

Serum from each stratum (smpd1^+/+^, smpd1^+/−^, p-smpd1^+/+^, t-smpd1^+/+^) at baseline, 24 hours, day 3 and 28 following sepsis induction was used for analysis of clinically used laboratory parameters of hepatobiliary damage and dysfunction (total-bilirubin T-Bil and γ-glutamyl-transferase γ-GT) using the clinical chemistry analyzer Fuji Dri-Chem 3500i (Sysmex, Leipzig, Germany) according to manufacturer’s instructions.

### Real-time PCR

Fresh frozen liver tissue samples (15–20 mg) were homogenized in 600 µL lysis buffer (RLT lysis puffer, supplemented with 1% β-mercaptoethanol; Qiagen, Hilden, Germany). RNA was isolated using RNeasy Mini Kit according to manufacturer’s instructions (Qiagen, Hilden, Germany). RNA concentration and integrity were analyzed using Nanodrop^TM^ spectrophometer and QIAxcel microcapillar electrophoresis apparatus and were followed by reverse transcription of 1.0 µg mRNA using standard conditions (Thermo Scientific, Germany)^57^. The expression profile of selected mRNA was measured using Rotor-Gene Q 2plex system. The following primer sequences were used:

ACTA2fw: tgatcaccatcggaaatgaa, rv: agaggtccttcctgatgtcaa

COL1A1 fw: caagaaccccaaggacaaga, rv: aggaaggtcagctggatgg

GAPDH fw: ctctgctcctcctgttcgac, rv: caatacgaccaaatccgttgac

Acta2fw: ccgagatctcaccgactacc, rv: tccagagcgacatagcacag

f4/80 fw: tttcctcgcctgcttcttc, rv: ccccgtctctgtattcaacc

Ccl2/mcp1 fw: aggtgtcccaaagaagctgtag, rv: aatgtatgtctggacccattcc

Tgfb fw: tatagcaacaattcctggcg, rv: tgctgtcacaagagcagtg

Tnfa fw: gtctactgaacttcggggtgat, rv: atgatctgagtgtgagggtctg

Il1b fw: gaagagcccatcctctgtga, rv: ttcatctcggagcctgtagtg

Csrp2 fw: gctacggaaagaagtatggacc, rv: ctcagtcagagttgtagactcc

Col1a1 fw: caaggtccttctggatcaagtg, rv: cctttatgcctctgtcaccttg

Col3a1 fw: acgtagatgaattgggatgcag, rv: gggttggggcagtctagtg

Mrp2 fw: aacttgggttgctccatga, rv: caggaccaggattttggattt

Actb fw: gctcttttccagccttcctt, rv: cggatgtcaacgtcacactt

### Histology

Representative liver samples from smpd1^+/+^, smpd1^+/−^, p-smpd1^+/+^, t-smpd1^+/+^ were fixed in neutral buffered formaldehyde 4% for at least 24 hours directly following harvesting. Samples were dehydrated in rising alcohol concentration and then embedded in paraffin. Livers were sliced into 3 µm sections and deparaffinized through xylene and reversed graded alcohol series. At least seven organs of each stratum were stained in Sirius Red, which was used to detect and to visualize extracellular matrix deposition. Two experienced researchers independently scored collagen deposition using a semi-quantitative scoring system in 20 randomly sampled high-power fields per animal semi-quantitatively in a blinded fashion as 0 (absent), 1 (mild), 2 (moderate), 3 (strong). Scores were accumulated for each animal.

### Statistics

For survival analysis, log rank test was used. Unpaired Mann-Whitney-U test (MWU) was performed to determine statistical differences between groups and over time. A level of p ≤ 0.05 was considered to be statistically significant. With respect to analyses of expression profile values log2 fold variations at least ± 1 were considered to be of biological significance.
